# Storage Location Allocation and Crane Scheduling Optimization in Automated Storage and Retrieval Systems with Dual I/O Ports

**DOI:** 10.3390/s26134194

**Published:** 2026-07-02

**Authors:** Zihang Yuan, Wenbin Zhang, Chunjiang Zhang

**Affiliations:** School of Mechanical Science and Engineering, Huazhong University of Science and Technology, Wuhan 430074, China; u202310397@hust.edu.cn (Z.Y.); wenbinzhang@hust.edu.cn (W.Z.)

**Keywords:** automatedstorage and retrieval system, scheduling optimization, repair-based constraint handling, memetic algorithm, sensor-informed scheduling

## Abstract

This paper addresses the challenge of integrated optimization for storage space allocation and crane scheduling in Automated Storage and Retrieval Systems (AS/RSs) under the complex constraints of dual I/O ports and Shared Storage strategies. The scheduling of multi-shuttle cranes in such environments constitutes a highly coupled NP-hard combinatorial optimization problem, where task assignment, sequencing, and dynamic storage location allocation must be solved simultaneously. To tackle this, this paper proposes an Elite-Driven Synchronized-Repair Memetic Algorithm (ED-SRMA) based on a sensor-informed total-travel-time objective formulated from horizontal and vertical crane displacements that can be measured by the crane drive sensors. The method combines a dual-layer representation, cross-layer synchronization, a problem-specific feasibility-repair operator, elite preservation, and budgeted local refinement. Numerical experiments were conducted on seven problem scales using 30 paired independent runs and a fixed evaluation budget. Compared with the corresponding dual-I/O operating mode without immediate location reuse, the shared-storage RS mode reduced the mean total travel time by 4.57–14.89%. ED-SRMA reduced the mean objective value by 5.48–39.59% relative to Base-GA and by 3.99–28.82% relative to PSO. For the representative 120×5 instance, the fitness-evaluation-based convergence analysis further shows an earlier objective-value reduction and continued improvement under the common evaluation budget. These results demonstrate the effectiveness, statistical significance, and consistency under the tested conditions of ED-SRMA for the investigated dual-I/O shared-storage scheduling problem.

## 1. Introduction

**Figure 1:**
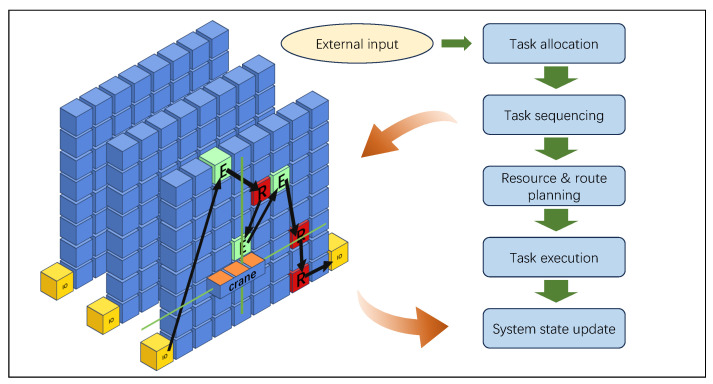
Illustration of the warehouse system workflow. Blue blocks represent storage racks; green and red blocks denote empty and retrieval locations, respectively; the orange block denotes the stacker crane; yellow blocks denote the I/O ports; and the arrows on the right indicate the scheduling and execution workflow.

An Automated Storage and Retrieval System (AS/RS) is the result of the integration of sensor technology [1], automated control technology [2], and Internet of Things (IoT) technology [3]. In this context, sensing and state acquisition provide the foundational layer for closed-loop scheduling, enabling the system to translate physical crane motion into quantifiable operational metrics. A typical AS/RS configuration consists of high-rise storage racks, input/output (I/O) stations, and automated stacker cranes that navigate aisles to perform tasks [4]. As illustrated in Figure 1, the fundamental workflow begins when storage or retrieval requests are generated by the Warehouse Management System (WMS). These tasks are then assigned to specific stacker cranes, which travel along the aisles to deposit items into a designated storage location or retrieve items for downstream processing [5]. The efficiency of this “Task Assignment-Path Planning-Execution” cycle is the key determinant of the overall throughput of the warehouse system [6].

Under the Industry 4.0 paradigm, the logistics sector is undergoing a significant transformation characterized by high-frequency, fragmented, and time-sensitive order structures [7,8,9]. Although intelligent warehousing presents a promising prospect for managing massive throughput with minimal human intervention, it also faces substantial operational challenges. The primary difficulty lies in scheduling multi-shuttle stacker cranes, a problem mathematically classified as an NP-hard combinatorial optimization task [10,11]. Unlike single-shuttle systems, multi-shuttle configurations introduce complex constraints related to capacity balancing and task sequencing [12]. Traditional heuristic methods often struggle to escape local optima [13,14] in these high-dimensional search spaces, creating a bottleneck where the physical capabilities of the hardware are limited by inefficient control algorithms [15].

**Figure 2:**
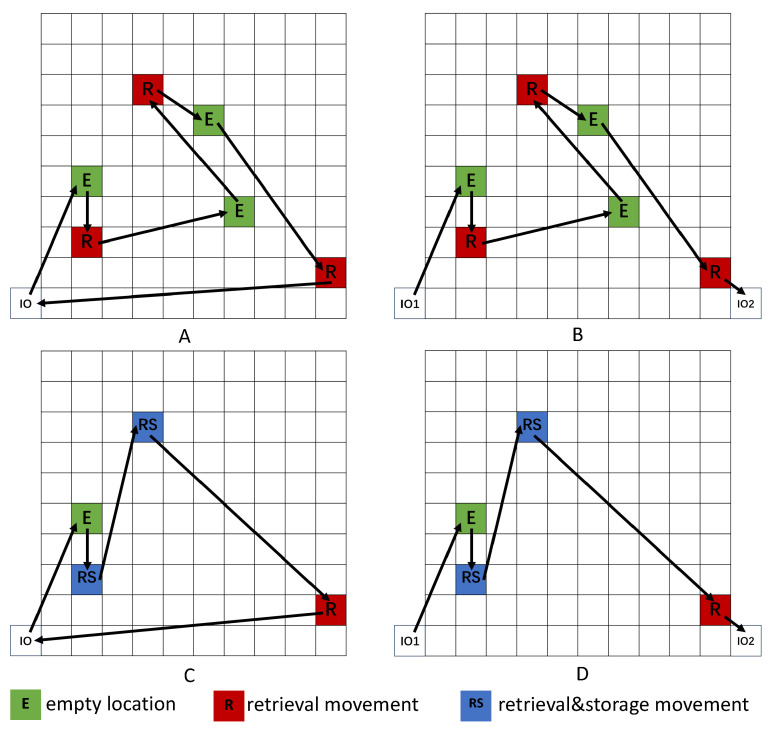
Schematic diagrams of storage (S), retrieval (R), and combined retrieval–storage (RS) operations under different topological layouts: (**A**) single-I/O non-shared storage; (**B**) dual-I/O non-shared storage; (**C**) single-I/O shared storage with RS operations; and (**D**) dual-I/O shared storage with RS operations. E, R, and RS denote an empty location, a retrieval movement, and a combined retrieval–storage movement, respectively.

The operating policy and I/O configuration further influence crane travel. Conventional non-shared storage separates storage (S) and retrieval (R) operations, as illustrated in Figure 2A. Under shared storage, a location released by a retrieval task can be immediately reused by a subsequent storage task. This retrieval–storage combination, referred to as an RS operation, eliminates the intermediate movement from the retrieval location to another empty location and can therefore reduce crane travel [16]. In addition, the dual-I/O configuration shown in Figure 2B,D allows the crane to select different entry and exit ports instead of returning to a fixed origin [17,18]. The combination of shared storage and dual-I/O flexibility provides additional opportunities for reducing empty travel, but it also couples storage-location allocation, within-trip operation sequencing, and inter-trip I/O-port selection.

Existing studies generally address only part of this coupled decision process. Storage-allocation studies commonly assume fixed command cycles or predetermined I/O configurations [19], whereas multi-I/O studies mainly optimize request sequencing or port assignment [20]. Research on double-end or multi-crane systems usually focuses on machine coordination and collision avoidance [21]. Consequently, limited attention has been given to the integrated scheduling of a single multi-shuttle stacker crane that performs interleaved storage and retrieval operations while the available empty locations and the starting I/O port evolve continuously across successive trips. Furthermore, generic evolutionary operators do not inherently preserve task uniqueness, full-load requirements, within-trip prefix feasibility, or consistency between task assignment and operation sequencing.

To address these limitations, this study formulates an integrated optimization model for a dual-I/O middle-aisle AS/RS with shared storage and a multi-shuttle stacker crane. The model jointly considers task grouping, storage/retrieval sequencing, dynamic empty-location evolution, RS operations, crane-capacity constraints, and inter-trip I/O continuity. Crane travel time is evaluated through a sensor-informed motion model based on horizontal and vertical displacement information derived from the crane drive sensors. This formulation provides a measurable interface between the analytical scheduling objective and the physical motion of the stacker crane.

The scientific significance of this study lies in incorporating dynamic location reuse and I/O-state propagation into a unified state-dependent scheduling model. Unlike models that evaluate crane trips independently, the proposed formulation continuously transfers the empty-location set and the ending I/O state from one trip to the next. From an engineering perspective, the model enables the physical flexibility provided by shared storage, multi-shuttle handling, and dual I/O ports to be converted into feasible reductions in crane travel time.

The main contributions of this study are summarized as follows:

- An integrated dual-I/O shared-storage scheduling model is developed to jointly represent task grouping, storage/retrieval sequencing, dynamic empty-location allocation, RS operations, multi-shuttle capacity, and inter-trip I/O continuity.
- A dual-layer solution representation is introduced to separate retrieval-task assignment from within-trip operation sequencing. A state-dependent decoding procedure continuously updates the crane position and the available empty-location set over all trips.
- An Elite-Driven Synchronized-Repair Memetic Algorithm (ED-SRMA) is proposed. Its synchronization and heuristic repair procedures restore cross-layer consistency and prefix-load feasibility after genetic variation, while elite preservation and budgeted local refinement improve the retention and refinement of promising feasible solutions.

The remainder of this paper is organized as follows: Section 2 reviews the related work on AS/RS scheduling and evolutionary algorithms. Section 3 formulates the integrated scheduling problem and presents an illustrative example. Section 4 describes the ED-SRMA framework, including its solution representation, state-dependent decoding, feasibility repair, cross-layer synchronization, evolutionary operators, and local refinement. Section 5 presents the numerical experiments and statistical analyses. Finally, Section 6 concludes the paper and outlines future research directions.

## 2. Literature Review

AS/RS research mainly concerns storage-location assignment, storage/retrieval sequencing, crane routing, and travel-time reduction [4,22,23]. These decisions become strongly coupled under shared storage, multiple I/O ports, and multi-shuttle operation because retrieval tasks release reusable locations, storage decisions change subsequent routes, and the ending port of one trip affects the next trip.

### 2.1. Storage-Location Allocation and Crane Scheduling

Tanaka and Araki studied crane routing under shared storage with separate input and output points [24], while Chen et al. jointly considered storage-location assignment and interleaving decisions [25]. Yang et al. further developed a variable-neighborhood-search method for integrated storage-location allocation and request sequencing in multi-shuttle AS/RS [16]. These studies showed that storage assignment and crane scheduling should be optimized jointly rather than sequentially. Effective encoding is crucial for evolutionary success in high-dimensional problems. Zhang et al. [26] introduced a multi-layer encoding method for integrated storage allocation and crane scheduling. The present study inherits this general integrated-optimization perspective but addresses a distinct dual-I/O shared-storage problem involving RS operations, dynamic empty-location evolution, and inter-trip I/O continuity. Accordingly, ED-SRMA introduces state-dependent decoding, cross-layer synchronization, feasibility repair, elite preservation, and budgeted local refinement.

Recent studies have extended this topic to storage reallocation and I/O-station configuration. Salah et al. proposed a two-step reallocation strategy that moves frequently requested items closer to the I/O station [27], and Alnahhal et al. examined the combined influence of storage reallocation and I/O-station location on throughput [19]. Rizqi et al. jointly considered storage assignment and I/O-point configuration from an energy-efficiency perspective [28]. However, these studies mainly assume offline reallocation, fixed command cycles, or predetermined I/O configurations and do not continuously update the available-location set throughout successive crane trips.

Multiple I/O points and multi-shuttle cranes provide additional routing flexibility but substantially enlarge the scheduling space. Song et al. developed a sorting method for large-scale requests in multi-I/O-depot AS/RSs [20], while Polten and Emde investigated task grouping and sequencing for multi-shuttle cranes [10]. Geng et al. considered routing and collision avoidance in a double-end system with two stacker cranes [18]. Yan et al. analyzed travel time and stacker-path scheduling in double-ended compact storage systems [29,30], and Hu et al. proposed a modified brain storm optimization algorithm for the same class of systems [31]. Xu et al. studied parallel-crane scheduling under shared storage [21].

Exact and network-based approaches have also been developed. Buckow et al. jointly investigated retrieval sequencing and I/O-point assignment and transformed several variants into traveling-salesman problems [32]. Ishikura et al. formulated multi-machine AS/RS scheduling through a time-expanded network [33]. Nevertheless, double-end and parallel-crane systems mainly emphasize machine coordination or collision avoidance, whereas multi-I/O retrieval models often assume retrieval-only operations, fixed storage locations, or single-load handling. They therefore do not directly represent interleaved storage and retrieval operations performed by a multi-shuttle crane under dynamic shared storage and continuous dual-I/O transitions.

### 2.2. Learning-Based Scheduling Methods

Recent studies have introduced data-driven and learning-based methods into storage-location allocation and crane scheduling. Antomarioni et al. developed a data-driven decision-support system for AS/RS item allocation [34], and Ekren and Arslan applied reinforcement learning to transaction scheduling in shuttle-based storage systems [35]. Rizqi et al. combined ensemble learning with metaheuristic optimization to predict the next request and determine the crane dwell point [36]. Rizqi and Chou subsequently integrated discrete-event simulation, deep reinforcement learning, and NSGA-II for dynamic and energy-aware crane scheduling [37]. Kouloughli et al. applied Q-learning to retrieval-time optimization but also reported reduced effectiveness under dynamic operating conditions [38]. Deep spatiotemporal learning has also been applied to transportation scheduling [39].

Learning-based methods are suitable for uncertain and dynamic environments, but their performance depends on training data, state representation, reward design, and parameter settings. More importantly, they do not inherently guarantee compliance with hard operational constraints, including crane capacity, within-trip load feasibility, dynamic location availability, and continuity between consecutive I/O ports. Consequently, structured combinatorial optimization remains necessary for the deterministic and strongly constrained problem considered in this study.

### 2.3. Research Gaps and Positioning of This Study

The literature reveals three main gaps. First, storage-location allocation, request sequencing, and I/O-port selection are commonly optimized separately, although these decisions are mutually dependent under shared storage. Second, existing models rarely propagate dynamic empty-location changes and I/O states across all crane trips; therefore, the immediate reuse of a retrieval location as an RS operation is not fully represented. Third, generic metaheuristics and learning methods do not directly preserve task uniqueness, full-load requirements, within-trip prefix feasibility, and cross-trip I/O continuity.

To address these gaps, this study formulates an integrated dual-I/O AS/RS model that simultaneously considers task grouping, storage/retrieval sequencing, dynamic empty-location evolution, RS operations, multi-shuttle capacity, and inter-trip I/O continuity. The proposed ED-SRMA employs a dual-layer representation to describe task assignment and operation sequencing, together with synchronization and heuristic repair operators to restore feasibility after genetic variation. Elite preservation and lightweight local search are incorporated to improve search efficiency, while displacement-sensor information is used to evaluate crane travel time. The proposed method therefore provides a feasible and transparent optimization framework for the integrated scheduling problem that is not directly addressed by existing storage-allocation, multi-I/O, double-end, or learning-based approaches.

## 3. Problem Description

This study investigates an AS/RS equipped with dual I/O ports and a single stacker crane. The crane operates within a two-dimensional aisle plane, enabling simultaneous movement along both horizontal and vertical axes. The system processes *n* storage requests and *n* retrieval requests, with both types of requests potentially assigned to either I/O port; notably, the two ports are not required to process equal numbers of requests.

A crane operation is organized into discrete trips. During trip *k*, the crane begins at port $IO_{a_{k}}$ and initially loads *m* unit loads. It then proceeds to visit a sequence of storage locations denoted by $\left(c_{k,1},c_{k,2},\ldots \right)$ within the rack. Throughout this traversal, storage and retrieval operations may be interleaved: when the crane encounters an empty location, it performs a storage move by depositing one unit load; conversely, when it visits an occupied retrieval location, it executes a retrieval operation, thereby releasing that location. As a result, the set of empty locations evolves dynamically: each retrieval releases a location to the empty set, while each storage move removes one location from it. Upon completing the assigned operations, the crane returns to an ending port $IO_{b_{k}}$ to unload the retrieved unit loads. Notably, I/O continuity is enforced: the ending port of trip *k* becomes the starting port of trip k+1.

To make the problem tractable, the following assumptions are adopted.

- Enforced full-load trips: each trip consists of exactly *m* storage moves and *m* retrieval operations (i.e., 2m tasks per trip), resulting in K=n/m trips; if *n* is not divisible by *m*, dummy tasks can be added to balance the workload. Storage and retrieval operations may be interleaved within a trip; however, a prefix feasibility condition is imposed to prevent capacity violations. Specifically, before any operation in a given process, the number of executed retrieval operations cannot exceed the number of executed storage moves.
- Known locations: All retrieval items and their storage locations are known a priori and are initially occupied. All other locations are initially empty, ensuring the availability of a feasible empty location for storage operations.
- Homogeneous handling times: The service times for a storage move (*S*), a retrieval operation (*R*), and a possible combined move (R/S) are assumed to be identical and constant. Therefore, they can be incorporated into a constant term and do not affect the comparison of alternative schedules.
- Single-device sequential execution: Only one crane is considered, and trips are executed sequentially; that is, congestion, blocking, and interference effects caused by additional devices are not accounted for.
- Constant speeds with simultaneous motion: The crane moves with constant speeds $v_{x}$ and $v_{y}$ along the two axes (in experiments, we set $v_{x}=v_{y}$). The travel time between two points is modeled using the Chebyshev time metric, which reflects that the travel duration is dominated by the slower axis-wise component.
- I/O continuity: The ending I/O port of trip *k* is used as the starting port of trip k+1, ensuring a continuous inter-trip trajectory and consistent time evaluation.

### 3.1. System Configuration and Sensing Interface

In a practical implementation, displacement sensors can be installed on the horizontal and vertical drives, while position sensors at the two I/O ports can be used to confirm crane arrival and cargo-exchange positions.

Potential for combined retrieval and storage (RS) moves: If a retrieval operation and a storage operation occur sequentially at the same location, the travel time between them is zero.

#### 3.1.1. Task Sets and I/O Assignment

Let P={1,2} denote the set of I/O ports. The storage-request set is $S=S^{\left(1\right)}\cup S^{\left(2\right)}$ and the retrieval-request set is $R=R^{\left(1\right)}\cup R^{\left(2\right)}$, where

(1) $$\begin{array}{cc} \; & \left\{\begin{array}{c} \left|S^{\left(1\right)}\right|=n_{s1}\\ \left|S^{\left(2\right)}\right|=n_{s2} \end{array}\right.\;n_{s1}+n_{s2}=n \end{array}$$

(2) $$\begin{array}{cc} \; & \left\{\begin{array}{c} \left|R^{\left(1\right)}\right|=n_{r1}\\ \left|R^{\left(2\right)}\right|=n_{r2} \end{array}\right.\;n_{r1}+n_{r2}=n \end{array}$$

(3) $$\begin{array}{cc} \; & n_{r1},n_{r2},n_{s1},n_{s2}\in Z\geq 0 \end{array}$$

#### 3.1.2. Storage Locations and Initial Empties

Let L be the set of storage locations with cardinality L=|L|. For experimental convenience and to guarantee sufficient initial empty locations, we consider a q×q grid of locations:

(4) $$q=\left\lfloor \sqrt{2n}\right\rfloor +1,\;L=q^{2}$$

thus L>2n. Let $L_{e}\left(0\right)\subseteq L$ be the set of empty locations at time zero. We assume all retrieval locations are known in advance and initially occupied by *n* items; hence, typically $|L_{e}\left(0\right)|\geq L-n>n$, which ensures feasibility even in a worst-case sequence.

### 3.2. Batch Definition and Capacity Constraints

The *k*-th crane trip is modeled as a batch $B_{k}$ consisting of a subset of storage tasks and retrieval tasks:

(5) $$B_{k}=\left(B_{k}^{S},B_{k}^{R}\right),\;B_{k}^{S}\subseteq S,\;B_{k}^{R}\subseteq R.$$

The capacity constraints are expressed as follows:

(6) $$|B_{k}^{S}\left|\leq m,\;\right|B_{k}^{R}\left|\leq m,\;\right|B_{k}^{S}\left|+\right|B_{k}^{R}|\leq 2m,\;\forall k.$$

Under the enforced full-load setting adopted in this study, exactly *m* storage tasks and *m* retrieval tasks are completed per trip [40]:

(7) $$|B_{k}^{S}\left|=\right|B_{k}^{R}|=m,\;p_{k}=\left|B_{k}\right|=2m,\;\forall k.$$

Accordingly, the number of trips is given by $K=n/m\in Z_{>0}$ (pseudo-tasks can be introduced otherwise). Finally, each task must be assigned to exactly one batch:

(8) $$\overset{K}{\underset{k=1}{⋃}}B_{k}=R\cup S,\;B_{k}\cap B_{k^{\prime }}=\emptyset \;\left(k\neq k^{\prime }\right).$$

### 3.3. Feasibility: Empty-Location and Load Constraints

#### 3.3.1. Empty-Location Feasibility (Global)

For any prefix of the first *k* trips, the cumulative number of stored items cannot exceed the initial empty location plus those released by retrievals:

(9) $$\sum_{j=1}^{k}|B_{j}^{S}\left|\leq \right|L_{e}\left(0\right)|+\sum_{j=1}^{k}\left|B_{j}^{R}\right|,\;\forall k.$$

#### 3.3.2. Load Feasibility (Within-Trip Prefix Constraint)

At the beginning of each trip, the crane carries *m* items. Executing a storage move decreases the onboard load by 1, whereas executing a retrieval operation increases it by 1. To prevent exceeding capacity *m*, the number of retrieval operations cannot exceed the number of storage moves in any prefix of the trip. Let $\pi_{k}=\left(\pi_{k,1},\ldots ,\pi_{k,2m}\right)$ be the operation-type sequence with $\pi_{k,j}\in \left\{S,R\right\}$; then

(10) $$\sum_{h=1}^{j}I\left[\pi_{k,h}=R\right]\leq \sum_{h=1}^{j}I\left[\pi_{k,h}=S\right],\;\forall k,\;\forall j=1,\ldots ,2m.$$

In the full-load setting, it is also common to enforce $\pi_{k,2m}=R$ so that the crane returns with *m* items.

### 3.4. Dual-I/O Continuity and Time Model

#### 3.4.1. I/O Continuity

Let $IO_{a_{k}}$ and $IO_{b_{k}}$ denote the start and end I/O ports of trip *k*, where $a_{k},b_{k}\in \left\{1,2\right\}$. The “end-port becomes next start-port” rule is captured by

(11) $$a_{k+1}=b_{k},\;k=1,\ldots ,K-1,$$

with a given initial port $a_{1}$ (e.g., $a_{1}=1$).

#### 3.4.2. Chebyshev Travel Time Measured by Displacement Sensors

This sensor-informed formulation improves the consistency between the analytical travel-time model and the physical motion behavior of the crane, thereby enhancing the practical interpretability of the optimization results. The distance between two points $u=(x_{u},y_{u})$ and $v=(x_{v},y_{v})$ on the shelf can be determined using the crane’s displacement sensors. Given the directional speeds of the crane $v_{x}$ and $v_{y}$, the travel time is formulated as follows:

(12) $$t\left(u,v\right)=\max\left(\frac{|x_{u}-x_{v}|}{v_{x}},\frac{|y_{u}-y_{v}|}{v_{y}}\right).$$

High-precision displacement sensors, which are installed on the crane’s motors in the X and Y directions, can also be used to provide feedback and to verify the effectiveness of scheduling in the actual system.

#### 3.4.3. Trip Time and Objective

Let $\left(c_{k,1},\ldots ,c_{k,p_{k}}\right)$ be the visited location sequence of trip *k* (each $c_{k,j}\in L$). The trip duration is

(13) $$T\left(B_{k}\right)=t\left(IO_{a_{k}},c_{k,1}\right)+\sum_{j=1}^{p_{k}-1}t\left(c_{k,j},c_{k,j+1}\right)+t\left(c_{k,p_{k}},IO_{b_{k}}\right),$$

Since a single crane executes trips sequentially, minimizing the total completion time is equivalent to minimizing the total travel time:

(14) $$\min\;T=\sum_{k=1}^{K}T\left(B_{k}\right).$$

### 3.5. Illustrative Example

A small instance is introduced to illustrate the interaction among shared storage, load feasibility, RS operations, and dual-I/O continuity. Consider a 4×4 rack with dual I/O ports located at ${\mathrm{IO}}_{1}=\left(1,0\right)$ and ${\mathrm{IO}}_{2}=\left(4,0\right)$. The system contains n=6 storage requests and n=6 retrieval requests. The crane capacity is m=3; therefore, the requests are completed in K=n/m=2 full-load trips.

The initial retrieval locations are

(15) $$\begin{array}{cccccc} R_{1} & =(2,1), & R_{2} & =(3,2), & R_{3} & =(4,1),\\ R_{4} & =(1,2), & R_{5} & =(2,4), & R_{6} & =(4,4), \end{array}$$

and all remaining rack locations constitute the initial empty-location set $L_{e}\left(0\right)$. One feasible schedule is

(16) $$\begin{array}{cc} B_{1}:\;{\mathrm{IO}}_{1}\; & \to S_{1}\left(1,1\right)\to R_{1}\left(2,1\right)\to S_{2}\left(2,1\right)\\ \; & \to R_{2}\left(3,2\right)\to S_{3}\left(3,2\right)\to R_{3}\left(4,1\right)\to {\mathrm{IO}}_{2},\\ B_{2}:\;{\mathrm{IO}}_{2}\; & \to S_{4}\left(4,1\right)\to R_{6}\left(4,4\right)\to S_{5}\left(4,4\right)\\ \; & \to R_{5}\left(2,4\right)\to S_{6}\left(2,4\right)\to R_{4}\left(1,2\right)\to {\mathrm{IO}}_{1}. \end{array}$$

For both trips, the operation-type sequence is (S,R,S,R,S,R). The cumulative numbers of storage and retrieval operations after each step are

(17) (1,0),(1,1),(2,1),(2,2),(3,2),(3,3),

respectively. Hence, the number of completed retrieval operations never exceeds the number of completed storage operations in any prefix. Starting with three unit loads, the onboard load evolves as

(18) 3→2→3→2→3→2→3,

which remains within the capacity interval [0,m].

In the first trip, the location released by $R_{1}$ is immediately reused by $S_{2}$, and the location released by $R_{2}$ is reused by $S_{3}$. These two pairs are therefore RS operations with zero intermediate travel time. After the first trip, the empty-location set becomes

(19) $$L_{e}\left(1\right)=\left(L_{e}\left(0\right)\setminus \left\{\left(1,1\right)\right\}\right)\cup \left\{\left(4,1\right)\right\}.$$

The newly released location (4,1) is then assigned to $S_{4}$ at the beginning of the second trip. This illustrates that storage-location availability must be propagated between consecutive trips rather than evaluated independently.

Assuming $v_{x}=v_{y}=1$, the travel times of the two trips obtained from Equation (12) are

(20) $$T(B_{1})=1+1+0+1+0+1+1=5,$$

(21) $$T(B_{2})=1+3+0+2+0+2+2=10.$$

Accordingly, the total travel time of this feasible schedule is

(22) $$T=T\left(B_{1}\right)+T\left(B_{2}\right)=15.$$

The ending port of $B_{1}$ is ${\mathrm{IO}}_{2}$, which is also the starting port of $B_{2}$, thereby satisfying the inter-trip I/O-continuity constraint. This example demonstrates that task sequencing, storage-location allocation, dynamic empty-location evolution, and I/O-port selection must be evaluated jointly.

## 4. Design and Implementation of ED-SRMA for Integrated Scheduling in Dual-I/O Middle-Aisle Warehouses

This section presents ED-SRMA for the integrated optimization of storage-location allocation and crane-trip scheduling in a dual-I/O middle-aisle AS/RS. The problem involves two coupled state transitions: inter-trip I/O continuity and the dynamic evolution of empty locations under shared-storage operations.

### 4.1. Overall Framework and Design Rationale of ED-SRMA

**Figure 3:**
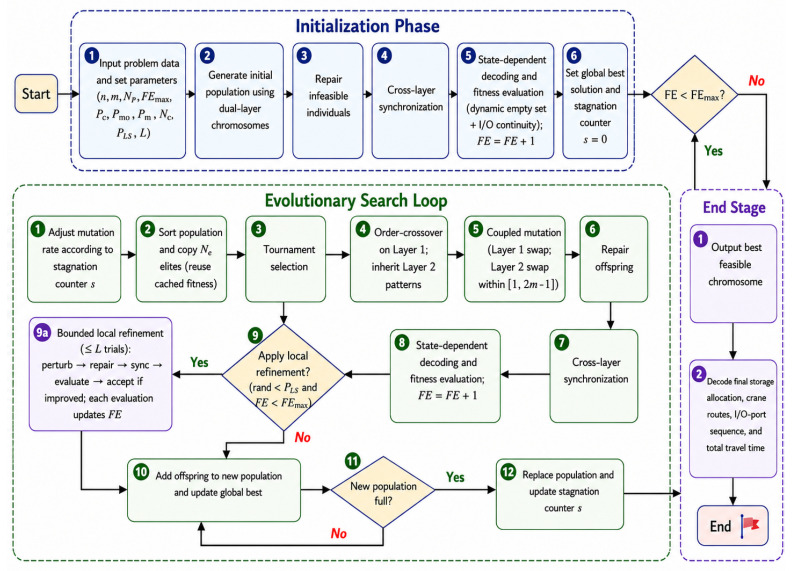
Overall framework of the proposed Elite-Driven Synchronized-Repair Memetic Algorithm.

Figure 3 presents the overall framework of ED-SRMA. The algorithm combines population-based search with problem-specific feasibility repair, cross-layer synchronization, elite preservation, adaptive mutation, and budgeted local refinement. The complete procedure is summarized in Algorithm 1, while the principal operators are described in the following subsections.

Algorithm 1 summarizes the complete ED-SRMA procedure. The algorithm initializes and evaluates a repaired dual-layer population, iteratively generates and repairs offspring, applies budgeted local refinement to selected feasible solutions, and updates the elite-driven population until the fitness-evaluation budget is exhausted. The representation, decoding, repair, evolutionary operators, and local-refinement procedure are detailed in the following subsections.

### 4.2. Dual-Layer Solution Representation and Population Initialization

The integrated optimization problem involves two coupled decisions: assigning retrieval tasks to crane trips and determining the execution order of storage and retrieval operations within each trip. To represent these decisions separately while preserving their relationship, each ED-SRMA individual is defined as a dual-layer chromosome

(23) $$X=\left\{X^{\left(1\right)},X^{\left(2\right)}\right\},$$

where $X^{\left(1\right)}$ represents the retrieval-task assignment and $X^{\left(2\right)}$ represents the within-trip operation sequence.
**Algorithm 1:** Overall procedure of the proposed ED-SRMA
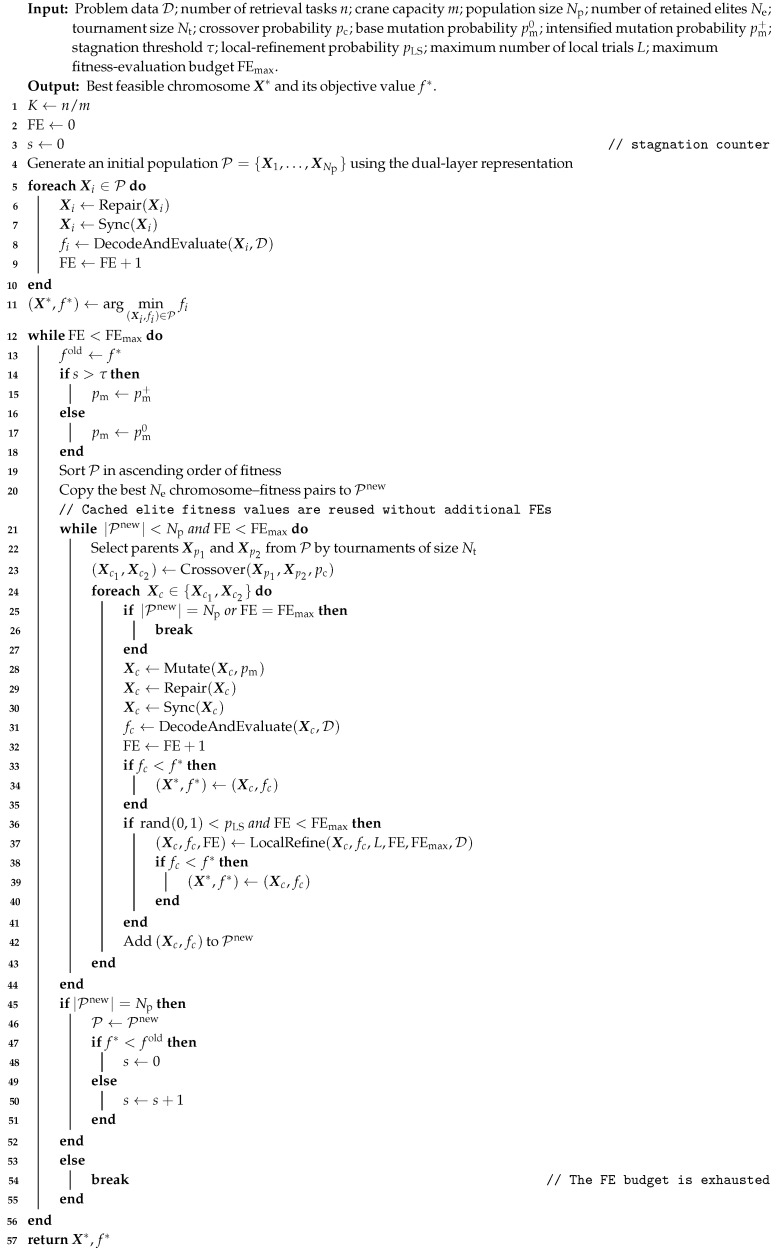


#### 4.2.1. Retrieval-Task Assignment Layer

Let *n* denote the number of retrieval tasks, *m* the crane capacity, and

(24) $$K=\frac{n}{m}$$

the number of trips under the full-load assumption. The first encoding layer is a K×m matrix,

(25) $$X^{\left(1\right)}={\left[x_{k,\ell }^{\left(1\right)}\right]}_{K\times m},\;x_{k,\ell }^{\left(1\right)}\in \left\{1,\ldots ,n\right\}.$$

The *k*th row contains the *m* retrieval tasks assigned to trip *k*. All entries in $X^{\left(1\right)}$ are distinct and satisfy

(26) $$\left\{x_{k,\ell }^{\left(1\right)}|k=1,\ldots ,K,\;\ell =1,\ldots ,m\right\}=\left\{1,\ldots ,n\right\}.$$

Therefore, each retrieval task is assigned to exactly one trip, with neither duplication nor omission.

The order of the retrieval identifiers within each row also determines their left-to-right placement in the retrieval positions of the second layer after cross-layer synchronization.

#### 4.2.2. Operation-Sequence Layer

The second encoding layer describes the sequence of storage and retrieval actions within each trip. Let

(27) H=max(K+1,m+1)

denote the number of symbolic storage genes provided in each row. The second layer is expressed as

(28) $$X^{\left(2\right)}={\left[x_{k,j}^{\left(2\right)}\right]}_{K\times (m+H)}.$$

For trip *k*, its initial gene set is

(29) $$G_{k}=\left\{R_{x_{k,1}^{\left(1\right)}},\ldots ,R_{x_{k,m}^{\left(1\right)}},E_{1},\ldots ,E_{H}\right\},$$

where $R_{i}$ represents retrieval task *i*, and $E_{j}$ is a symbolic storage-action gene.

A retrieval gene is associated with a known retrieval location. In contrast, a storage gene does not identify a fixed rack location. Its physical target is selected during state-dependent decoding from the current empty-location set. Thus, the symbolic index *j* distinguishes storage genes in the chromosome but does not correspond to a physical location.

Only the first 2m positions of each row constitute the effective execution window:

(30) $$W_{k}=\left(x_{k,1}^{\left(2\right)},x_{k,2}^{\left(2\right)},\ldots ,x_{k,2m}^{\left(2\right)}\right).$$

After feasibility repair, this window contains exactly *m* retrieval genes and *m* storage genes. The remaining H−m positions are reserve positions and are not decoded during fitness evaluation.

#### 4.2.3. Population Initialization

For each individual, the first encoding layer is initialized by randomly permuting the *n* retrieval-task identifiers and reshaping the resulting permutation into a K×m matrix. For every trip, the corresponding retrieval genes are then combined with the *H* symbolic storage genes, and the resulting m+H genes are randomly permuted to form one row of $X^{\left(2\right)}$.

The initialization of row *k* can be expressed as

(31) $$X_{k,:}^{\left(2\right)}=\pi_{k}\left(R_{x_{k,1}^{\left(1\right)}},\ldots ,R_{x_{k,m}^{\left(1\right)}},E_{1},\ldots ,E_{H}\right),$$

where $\pi_{k}\left(\cdot \right)$ denotes an independently generated random permutation.

Because random permutations do not necessarily satisfy the operational constraints, each initialized individual is subsequently processed by the feasibility-repair and cross-layer synchronization procedures:

(32) $$X\leftarrow \mathrm{Sync}\left(\mathrm{Repair}\left(X\right)\right).$$

The repair operator establishes a feasible storage–retrieval pattern, while the synchronization operator ensures that the retrieval identifiers in the second layer are consistent with the corresponding row of the first layer. Their detailed implementation is presented in Section 4.4.

#### 4.2.4. Illustrative Encoding Example

Consider an instance with n=6 retrieval tasks and a crane capacity of m=2. The number of trips is K=3, and the number of symbolic storage genes is H=max(4,3)=4. A possible first-layer chromosome is

(33) $$X^{\left(1\right)}=\left[\begin{array}{cc} 4 & 1\\ 6 & 2\\ 5 & 3 \end{array}\right].$$

Accordingly, retrieval tasks {4,1}, {6,2}, and {5,3} are assigned to trips 1, 2, and 3, respectively.

After row-wise randomization, feasibility repair, and synchronization, a corresponding second-layer chromosome may be written as

(34) $$X^{\left(2\right)}=\left[\begin{array}{cccccc} E_{4} & E_{3} & R_{4} & R_{1} & E_{2} & E_{1}\\ E_{2} & R_{6} & E_{4} & R_{2} & E_{1} & E_{3}\\ E_{3} & E_{1} & R_{5} & R_{3} & E_{4} & E_{2} \end{array}\right].$$

Since 2m=4, only the first four positions of each row are executed. For example, the effective sequence of trip 2 is

(35) $$W_{2}=\left(E_{2},R_{6},E_{4},R_{2}\right).$$

The last two storage genes in the same row are reserve genes and do not participate in the current trip.

This representation separates retrieval-task assignment from the storage–retrieval operations pattern while retaining their correspondence through synchronization. The conversion of an effective sequence into physical storage locations, retrieval locations, crane movements, and I/O-port decisions is described in the following subsection.

### 4.3. State-Dependent Decoding and Fitness Evaluation

After feasibility repair and cross-layer synchronization, each chromosome is decoded into a continuous crane schedule. The decoding process is state dependent because the starting I/O port and the available empty locations of a trip depend on the operations completed previously. Accordingly, trips cannot be evaluated independently.

#### 4.3.1. I/O-Linked Trip Continuity

Let $P=\{{\mathrm{IO}}_{1},{\mathrm{IO}}_{2}\}$ denote the set of I/O ports. The starting and ending ports of trip *k* are denoted by $p_{k}^{\mathrm{start}}$ and $p_{k}^{\mathrm{end}}$, respectively. Inter-trip continuity is enforced by

(36) $$p_{1}^{\mathrm{start}}=p^{0},\;p_{k}^{\mathrm{start}}=p_{k-1}^{\mathrm{end}},\;k=2,\ldots ,K,$$

where $p^{0}$ is the prescribed initial crane position. In the experiments, $p^{0}={\mathrm{IO}}_{1}$.

After the 2m actions of trip *k* have been decoded, its ending port is selected according to

(37) $$p_{k}^{\mathrm{end}}=\arg\min_{p\in P}t\left(x_{k}^{\mathrm{last}},p\right),$$

where $x_{k}^{\mathrm{last}}$ is the final visited location and t(·,·) is the Chebyshev travel-time function defined in Section 3.4. When both ports produce the same return time, ${\mathrm{IO}}_{1}$ is selected to ensure deterministic evaluation.

#### 4.3.2. Dynamic Empty-Location Set

Let $E^{0}$ be the initial set of empty locations. At the beginning of chromosome evaluation,

(38) $$E\leftarrow E^{0}.$$

The set is updated continuously over all trips and is not reinitialized between consecutive trips.

For an ordinary retrieval operation $R_{i}$, the crane visits the known retrieval location $r_{i}$, after which this location becomes available:

(39) $$E\leftarrow E\cup \{r_{i}\}.$$

For an ordinary storage action, its physical target is selected from the current empty-location set using

(40) $$e^{*}=\arg\min_{e\in E}t\left(x,e\right),$$

where x is the current crane position. The selected location is then removed:

(41) $$E\leftarrow E\setminus \{e^{*}\}.$$

If several locations have the same minimum travel time, the first location in the fixed empty-set ordering is selected.

An adjacent retrieval–storage pair,

(42) $$\left(R_{i},E_{q}\right),$$

is decoded as an RS operation. The storage action immediately reuses the location released by $R_{i}$; therefore, both genes are mapped to $r_{i}$ and

(43) $$E_{\mathrm{after}}=E_{\mathrm{before}}.$$

The movement between the retrieval and storage actions is zero.

#### 4.3.3. Decoding and Fitness Calculation

For trip *k*, only the effective window

(44) $$W_{k}=X_{k,1:2m}^{\left(2\right)}$$

is decoded. Let $c_{k,1},\ldots ,c_{k,2m}$ denote the physical locations obtained from the genes in this window. The travel time of trip *k* is

(45) $$\begin{array}{cc} T_{k}= & t\left(p_{k}^{\mathrm{start}},c_{k,1}\right)+\sum_{j=1}^{2m-1}t\left(c_{k,j},c_{k,j+1}\right)\\ \; & +t\left(c_{k,2m},p_{k}^{\mathrm{end}}\right). \end{array}$$

The chromosome fitness is the total travel time

(46) $$F\left(X\right)=\sum_{k=1}^{K}T_{k}.$$

The complete procedure is summarized in Algorithm 2.
**Algorithm 2:** State-dependent decoding and fitness evaluation
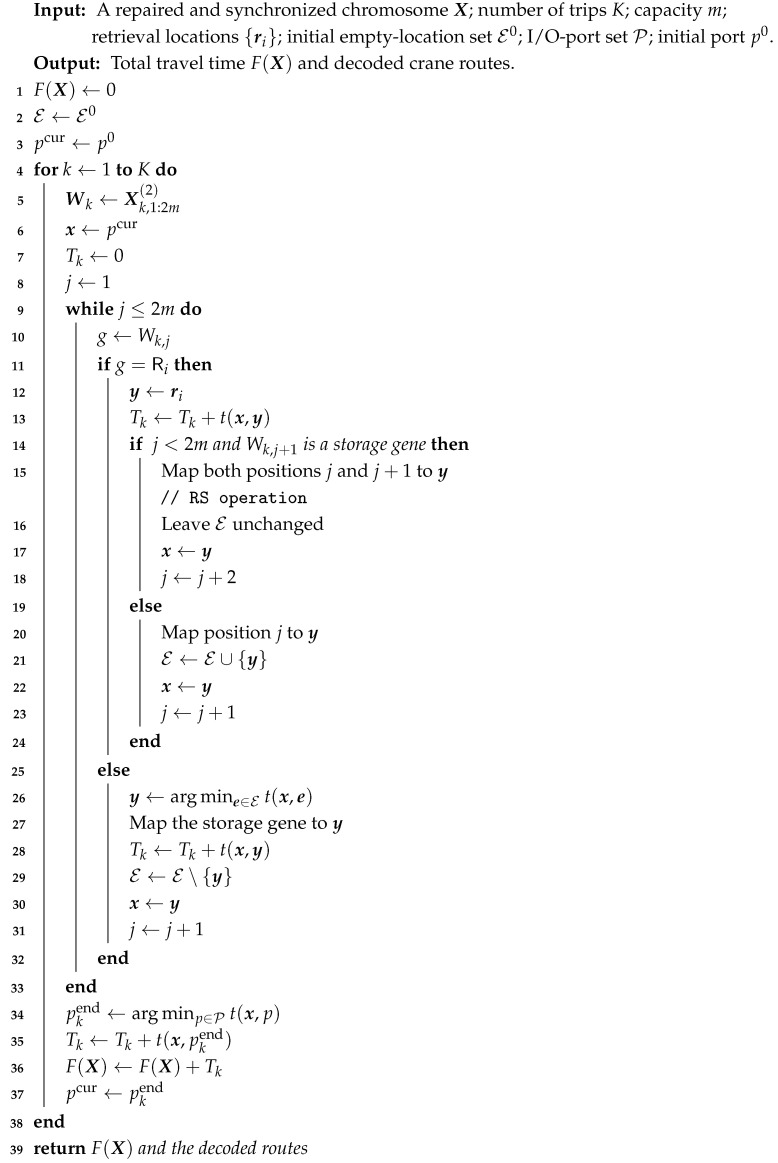


#### 4.3.4. Illustrative Decoding Example

Consider the effective sequence introduced in Section 4.2:

(47) $$W_{2}=\left(E_{2},R_{6},E_{4},R_{2}\right).$$

Let the starting port be ${\mathrm{IO}}_{1}=\left(1,0\right)$, the second port be ${\mathrm{IO}}_{2}=\left(5,0\right)$, and

(48) $$r_{6}=\left(3,2\right),\;r_{2}=\left(4,1\right).$$

Assume that the current empty-location set is

(49) E={(2,1),(1,3),(4,4)}.

The sequence is decoded as follows:

1. $E_{2}$ selects (2,1), the nearest empty location to ${\mathrm{IO}}_{1}$. The location is then removed from E.
2. $R_{6}$ is immediately followed by $E_{4}$. The pair is therefore decoded as an RS operation at (3,2), and the empty-location set remains unchanged.
3. $R_{2}$ is decoded at (4,1). Because it is not followed by a storage gene, (4,1) is added to E.
4. From (4,1), ${\mathrm{IO}}_{2}$ is selected as the ending port. It subsequently becomes the starting port of trip 3.

The corresponding physical visit sequence is

(50) $$\left[(2,1),(3,2),(3,2),(4,1)\right],$$

where the repeated coordinate represents the zero-distance movement within the RS operation. The resulting trip time is

(51) $$\begin{array}{cc} T_{2}= & t\left((1,0),(2,1)\right)+t\left((2,1),(3,2)\right)\\ \; & +t\left((3,2),(3,2)\right)+t\left((3,2),(4,1)\right)+t\left((4,1),(5,0)\right)\\ = & 1+1+0+1+1=4. \end{array}$$

This example shows how the symbolic storage genes are converted into physical storage locations according to the current system state. The feasibility of the operation sequence itself, including the execution-window structure and prefix load constraint, is ensured by the repair and synchronization procedures described in Section 4.4.

### 4.4. Feasibility Repair and Cross-Layer Synchronization

Genetic variation may disrupt the operation structure of the second encoding layer or make the retrieval identifiers inconsistent with the first layer. Instead of introducing penalty coefficients, ED-SRMA restores each candidate solution through two consecutive procedures:

(52) $$X\leftarrow \mathrm{Sync}\left(\mathrm{Repair}\left(X\right)\right).$$

The repair operator first establishes a feasible storage–retrieval pattern in each effective execution window. Cross-layer synchronization then assigns the retrieval identifiers represented by the first layer to the repaired retrieval positions in the second layer.

#### 4.4.1. Feasibility Conditions

For trip *k*, let

(53) $$W_{k}=\left(x_{k,1}^{\left(2\right)},\ldots ,x_{k,2m}^{\left(2\right)}\right)$$

denote the effective execution window. A feasible window must satisfy the following conditions.

First, the window contains exactly *m* storage genes and *m* retrieval genes:

(54) $$\begin{array}{cc} \sum_{j=1}^{2m}I\left(x_{k,j}^{\left(2\right)}\;\mathrm{is}\;E\right)\; & =m,\\ \sum_{j=1}^{2m}I\left(x_{k,j}^{\left(2\right)}\;\mathrm{is}\;R\right)\; & =m. \end{array}$$

Second, the first action is a storage action and the terminal action is a retrieval operation:

(55) $$x_{k,1}^{\left(2\right)}\in E,\;x_{k,2m}^{\left(2\right)}\in R.$$

Third, every prefix of the window satisfies the crane-capacity constraint:

(56) $$\sum_{t=1}^{j}I\left(x_{k,t}^{\left(2\right)}\;\mathrm{is}\;R\right)\leq \sum_{t=1}^{j}I\left(x_{k,t}^{\left(2\right)}\;\mathrm{is}\;E\right),\;j=1,\ldots ,2m.$$

Since the crane begins each trip with *m* inbound unit loads, this condition prevents its onboard load from exceeding capacity after a retrieval operation. Retrieval-task completeness and uniqueness are separately guaranteed by the permutation structure of the first encoding layer.

#### 4.4.2. Four-Stage Repair Procedure

The repair operator processes each row of the second layer through four stages.

*Stage 1: initial-action correction.* If the first position is occupied by a retrieval gene, it is exchanged with the rightmost storage gene in the row. This ensures that the trip begins with a storage action.

*Stage 2: execution-window alignment.* Any retrieval gene located outside the effective window [1,2m] is exchanged with a storage gene inside the window. The operation is repeated until all *m* retrieval genes are contained in the effective window. Consequently, the window contains exactly *m* storage and *m* retrieval operations.

*Stage 3: terminal-action correction.* If position 2m contains a storage gene, it is exchanged with the rightmost retrieval gene preceding that position.

*Stage 4: prefix-load correction.* The repaired window is scanned from left to right. When a retrieval operation at position *j* would violate Equation (56), the rightmost available storage gene in positions j,…,2m−1 is moved immediately before the violating retrieval operation. The scan is then restarted until all prefixes are feasible. Position 2m is excluded from this search so that the terminal retrieval condition is preserved.

This procedure modifies only the operation arrangement. The definitive retrieval-task identifiers are assigned afterward by cross-layer synchronization.

#### 4.4.3. Cross-Layer Synchronization

Let the retrieval positions in the repaired window of trip *k* be

(57) $$Q_{k}=\left(q_{k,1},q_{k,2},\ldots ,q_{k,m}\right),\;q_{k,1}<\cdots <q_{k,m}.$$

The synchronization operator replaces the retrieval identifiers at these positions according to row *k* of the first encoding layer:

(58) $$x_{k,q_{k,\ell }}^{\left(2\right)}\leftarrow R_{x_{k,\ell }^{\left(1\right)}},\;\ell =1,\ldots ,m.$$

The storage–retrieval pattern remains unchanged; only the retrieval identifiers are updated. Therefore, synchronization preserves the feasibility established by the repair operator while ensuring that both encoding layers represent the same trip-level task assignment.

The complete repair and synchronization procedure is summarized in Algorithm 3.
**Algorithm 3:** Feasibility repair and cross-layer synchronization
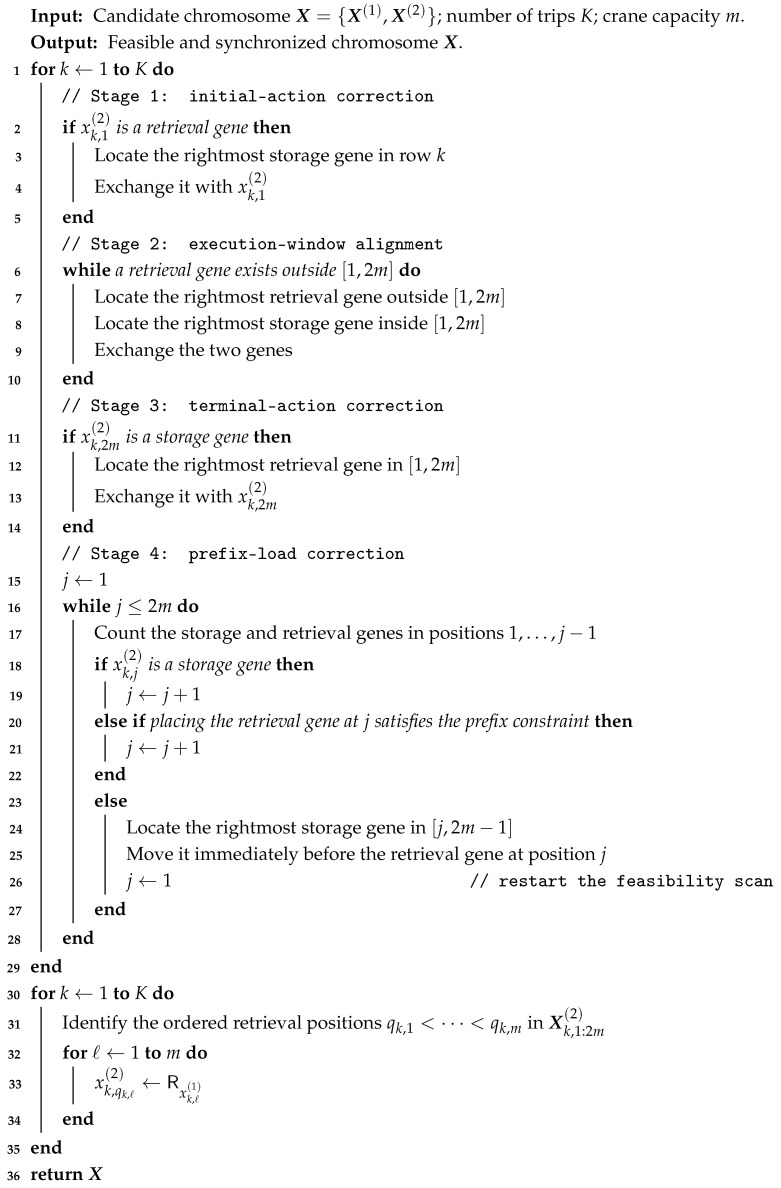


#### 4.4.4. Illustrative Repair and Synchronization Example

Consider the first-layer assignment introduced in Section 4.2:

(59) $$X_{1,:}^{\left(1\right)}=\left[4,\;1\right],$$

with m=2. Suppose that genetic variation produces the following second-layer row:

(60) $${\tilde{X}}_{1,:}^{\left(2\right)}=\left[R_{4},R_{1},E_{1},E_{2},E_{3},E_{4}\right].$$

The effective window consists of the first four positions. The row is infeasible because it begins with a retrieval operation and one retrieval gene lies outside the execution window.

Stage 1 exchanges the first gene with the rightmost storage gene:

(61) $$\left[E_{4},R_{1},E_{1},E_{2},E_{3},R_{4}\right].$$

Stage 2 moves the retrieval gene at position 6 into the execution window by exchanging it with the rightmost storage gene in that window:

(62) $$\left[E_{4},R_{1},E_{1},R_{4},E_{3},E_{2}\right].$$

The terminal position of the effective window is already a retrieval gene, and all prefixes satisfy Equation (56); therefore, no further structural modification is required.

The retrieval positions are 2 and 4. Synchronization then writes the first-layer assignment [4,1] into these positions:

(63) $$\begin{array}{cc} \; & \left[E_{4},R_{1},E_{1},R_{4},E_{3},E_{2}\right]\\ \; & \;\overset{\mathrm{Sync}}{\to }\left[E_{4},R_{4},E_{1},R_{1},E_{3},E_{2}\right]. \end{array}$$

The resulting effective sequence is feasible and consistent with the first-layer task assignment. It can therefore be evaluated using the state-dependent decoding procedure described in Section 4.3.

The same repair–synchronization sequence is applied after crossover, mutation, and every neighborhood perturbation. The evolutionary operators that generate these candidate solutions are described in the following subsection.

### 4.5. Evolutionary Operators and Elite-Driven Population Update

After initialization and fitness evaluation, ED-SRMA evolves the population through tournament selection, order crossover, coupled mutation, and elite-driven replacement. The evolutionary operators generate candidate solutions, which are subsequently processed by the repair and synchronization procedure described in Section 4.4.

#### 4.5.1. Tournament Selection

To select one parent, $N_{t}$ individuals are sampled uniformly without replacement from the current population. The individual with the smallest objective value is selected:

(64) $$X^{p}=\arg\min_{X\in T}F\left(X\right),\;\left|T\right|=N_{t},$$

where T denotes the tournament set. Two tournaments are conducted independently to obtain a pair of parents. The tournament size used in the experiments is reported in Section 5.1.

#### 4.5.2. Order Crossover

Crossover is applied with probability $p_{c}$. Because the first encoding layer is a permutation of all retrieval-task identifiers, it is flattened into a vector before applying order crossover (OX). Let

(65) $$v^{p_{1}}=\mathrm{vec}\left(X^{\left(1\right),p_{1}}\right),\;v^{p_{2}}=\mathrm{vec}\left(X^{\left(1\right),p_{2}}\right)$$

denote the two parent permutations. Two cut positions a<b are selected randomly. For the first offspring, the segment $v_{a:b}^{p_{1}}$ is retained, and the remaining positions are filled in the order in which unused task identifiers appear in $v^{p_{2}}$. The second offspring is generated symmetrically:

(66) $$\left(v^{c_{1}},v^{c_{2}}\right)=\mathrm{OX}\left(v^{p_{1}},v^{p_{2}};a,b\right).$$

The offspring vectors are then reshaped into K×m matrices. This operator preserves the uniqueness and completeness of the retrieval-task identifiers.

The second-layer operation pattern of each offspring is initially inherited from its corresponding parent. After crossover and mutation, cross-layer synchronization updates the retrieval identifiers in the second layer according to the modified first-layer assignment.

#### 4.5.3. Coupled Mutation

Mutation is applied independently to the two encoding layers with probability $p_{m}$.

For the first layer, two positions are selected from the flattened retrieval-task permutation and exchanged:

(67) $$\left(v_{u}^{c},v_{v}^{c}\right)\leftarrow \left(v_{v}^{c},v_{u}^{c}\right),\;u\neq v.$$

This operation changes both trip assignment and retrieval order while preserving the permutation property.

For the second layer, one trip is selected randomly, followed by two positions from

(68) {1,…,2m−1}.

The two genes are exchanged. Position 2m is excluded so that the prescribed terminal retrieval position is not directly disturbed:

(69) $$\left(x_{k,u}^{\left(2\right)},x_{k,v}^{\left(2\right)}\right)\leftarrow \left(x_{k,v}^{\left(2\right)},x_{k,u}^{\left(2\right)}\right),\;u,v\in \left\{1,\ldots ,2m-1\right\}.$$

Although this restriction preserves the terminal position, other feasibility conditions may still be violated. Therefore, every mutated offspring is repaired and synchronized before fitness evaluation.

#### 4.5.4. Adaptive Mutation

A stagnation counter *s* records the number of consecutive completed generations without improvement in the global best objective value. The mutation probability is adjusted according to

(70) $$p_{m}=\left\{\begin{array}{cc} p_{m}^{0}, & s\leq \tau ,\\ p_{m}^{+}, & s>\tau , \end{array}\right.\;p_{m}^{+}>p_{m}^{0},$$

where $p_{m}^{0}$ is the base mutation probability and τ is the stagnation threshold. When an improved global best solution is obtained, the counter is reset to zero. Otherwise, it is increased by one after the generation is completed. This mechanism introduces stronger perturbations only when the search remains stagnant.

#### 4.5.5. Elite-Driven Population Update

At the beginning of each generation, the current population is sorted in ascending order of fitness. The best $N_{e}$ individuals are copied directly to the next population:

(71) $$P^{\mathrm{new}}\leftarrow \left\{X_{\left(1\right)},\ldots ,X_{\left(N_{e}\right)}\right\},$$

where

(72) $$F\left(X_{\left(1\right)}\right)\leq \cdots \leq F\left(X_{\left(N_{p}\right)}\right).$$

Their previously calculated fitness values are retained and are not evaluated again. This avoids unnecessary consumption of the fitness-evaluation budget.

The remaining positions in $P^{\mathrm{new}}$ are filled with repaired and synchronized offspring generated by the preceding operators. Each offspring is evaluated once and may subsequently undergo the budgeted memetic refinement described in Section 4.6. When the new population is complete, it replaces the current population:

(73) $$P\leftarrow P^{\mathrm{new}}.$$

The global best solution is updated whenever an offspring or a locally refined solution obtains a smaller objective value. The evolutionary process continues until the common fitness-evaluation budget is exhausted. The common selection, crossover, and adaptive-mutation parameters, together with the controlled ablation settings for elite preservation and local refinement, are reported in Section 5.1.

### 4.6. Budgeted Memetic Local Refinement

After an offspring has been repaired, synchronized, and evaluated, ED-SRMA may apply a lightweight local-refinement procedure to further improve its objective value. The procedure uses small neighborhood moves, a strict improvement rule, and a limited fitness-evaluation budget. It therefore complements the population-based search without introducing an unrestricted computational overhead.

#### 4.6.1. Triggering Rule and Local Budget

For each evaluated offspring X, local refinement is activated with probability

(74) $$\mathrm{Pr}\;\left(\mathrm{LocalRefine}(X)\right)=p_{\mathrm{LS}}.$$

Once activated, at most *L* neighboring solutions are evaluated. The procedure also terminates immediately when the remaining global fitness-evaluation budget is exhausted:

(75) $$N_{\mathrm{trial}}\leq \min\left\{L,\;{\mathrm{FE}}_{\max}-\mathrm{FE}\right\}.$$

The values of $p_{\mathrm{LS}}$ and *L* used in the experiments are reported in Section 5.1.

#### 4.6.2. Neighborhood Structures

Two neighborhoods are defined according to the dual-layer representation.

For the first layer, the task-assignment neighborhood $N_{1}\left(X\right)$ is generated by flattening $X^{\left(1\right)}$, selecting two distinct positions *u* and *v*, and exchanging their retrieval-task identifiers:

(76) $$\left(x_{u}^{\left(1\right)},x_{v}^{\left(1\right)}\right)\leftarrow \left(x_{v}^{\left(1\right)},x_{u}^{\left(1\right)}\right).$$

The resulting vector is reshaped into the original K×m matrix. This move preserves task completeness and uniqueness while modifying the trip assignment or the retrieval order.

For the second layer, the operation-sequence neighborhood $N_{2}\left(X\right)$ is generated by selecting one trip *k* and two distinct positions

(77) u,v∈{1,…,2m−1},u≠v,

and exchanging the corresponding genes:

(78) $$\left(x_{k,u}^{\left(2\right)},x_{k,v}^{\left(2\right)}\right)\leftarrow \left(x_{k,v}^{\left(2\right)},x_{k,u}^{\left(2\right)}\right).$$

Position 2m is excluded to preserve the terminal retrieval position. When m=1, this neighborhood cannot select two distinct positions and only $N_{1}$ is used.

At each trial, one of the two neighborhoods is selected with equal probability when both are available. Only one swap operation is applied, which limits the difference between the current solution and its neighbor.

#### 4.6.3. Feasibility Restoration and Acceptance Rule

A neighborhood move may disturb the operation pattern or the correspondence between the two encoding layers. Therefore, every generated neighbor $X^{\prime }$ is processed according to

(79) $$X^{\prime }\leftarrow \mathrm{Sync}\left(\mathrm{Repair}\left(X^{\prime }\right)\right),$$

before its objective value is calculated.

Let X denote the current solution and $X^{\prime }$ its feasible neighbor. The neighbor is accepted only if

(80) $$F\left(X^{\prime }\right)<F\left(X\right).$$

Otherwise, the current solution remains unchanged. When a neighbor is accepted, it becomes the reference solution for the subsequent trial. Thus, several successive improvements may be obtained within one invocation of the local procedure.

The complete implementation is given in Algorithm 4.
**Algorithm 4:** Budgeted memetic local refinement
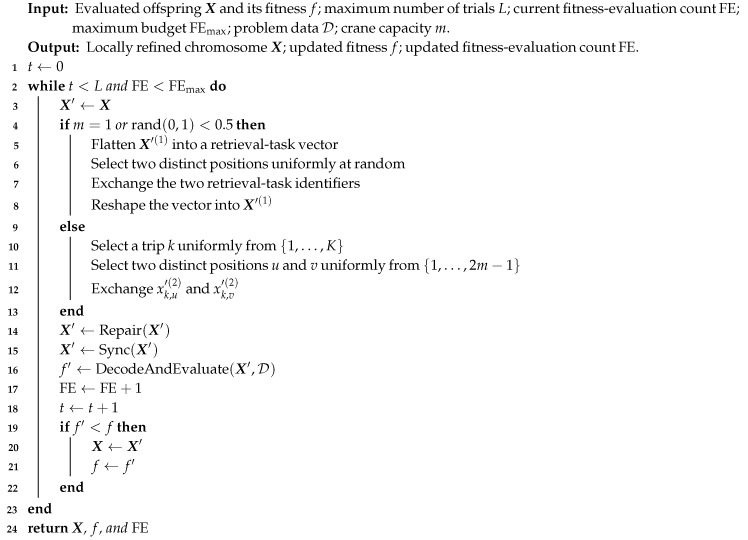


Every neighborhood evaluation in Algorithm 4 is counted as one fitness evaluation. Rejected neighbors therefore consume the same evaluation budget as accepted neighbors. The procedure returns the best solution obtained along the accepted sequence and passes it to the population-update process described in Section 4.5. Together with the elite-driven update, this bounded local improvement constitutes the memetic component of ED-SRMA.

## 5. Numerical Experiments and Performance Analysis

This section evaluates the proposed operating model and ED-SRMA through an RS-mode comparison, a controlled ablation study, an external comparison with PSO, FE-based convergence analysis, and shuttle-capacity sensitivity analysis. All comparisons use paired runs and a common fitness-evaluation budget.

### 5.1. Experimental Setup and Fair Comparison Protocol

#### 5.1.1. Warehouse Configuration and Instance Generation

The experiments consider a dual-I/O middle-aisle AS/RS with one stacker crane. Let *n* denote the number of retrieval tasks; an equal number of storage tasks is considered. For each problem instance, the rack is represented by a q×q square grid, where

(81) $$q=\left\lfloor \sqrt{2n}\right\rfloor +1,\;L=q^{2}.$$

Since L>2n, the *n* retrieval locations are initially occupied and the remaining L−n locations are initially empty. The initial vacancy ratio is therefore greater than 50%, which provides sufficient empty locations for shared-storage operations.

Retrieval locations are sampled without replacement from the rack grid, and their coordinates are assumed to be known before scheduling. The dual I/O ports are located at

(82) $${\mathrm{IO}}_{1}=\left(1,0\right),\;{\mathrm{IO}}_{2}=\left(q,0\right).$$

The horizontal and vertical crane velocities are set to $v_{x}=v_{y}=1.0$. Travel time is calculated using the Chebyshev metric defined in Section 3.4.2. Consequently, the reported objective values represent the total crane travel time under normalized motion speeds.

All algorithms were implemented in MATLAB R2024a and executed on the same computing platform. No parallel computation was used during the formal experiments.

#### 5.1.2. Test Instances and Compared Methods

Seven problem scales were considered:

(83) $$\begin{array}{cc} n\times m\in \{ & 12\times 3,\;24\times 2,\;48\times 6,\;60\times 3,\\ \; & 96\times 4,\;120\times 5,\;144\times 4\}, \end{array}$$

where *n* denotes the number of retrieval requests and *m* denotes the crane capacity. These instances cover different task sizes and within-trip sequencing complexities.

Three groups of experiments were conducted. First, the Dual-I/O No-RS and Dual-I/O RS operating modes were compared using the same ED-SRMA optimizer to evaluate the effect of immediate location reuse. Second, Base-GA, ED-GA, SR-MA, and ED-SRMA were compared in the ablation study. Their mechanism configurations are summarized in Table 1. Third, particle swarm optimization (PSO) was used as an external metaheuristic benchmark under the same fitness-evaluation budget. The implementation and representation of PSO are described in Section 5.4.

For the shuttle-capacity sensitivity experiment, the number of retrieval requests was fixed at n=60, while

(84) m∈{1,2,3,4,5,6,10}.

#### 5.1.3. Algorithm Parameter Settings

To ensure a controlled ablation study, the four genetic variants use the same population initialization, tournament selection, order crossover, coupled mutation, adaptive-mutation rule, feasibility repair, cross-layer synchronization, state-dependent decoding, and termination criterion. Their common parameters are

(85) $$\begin{array}{cccccc} N_{p} & =30, & N_{t} & =3, & p_{c} & =0.80,\\ p_{m}^{0} & =0.05, & p_{m}^{+} & =0.20, & \tau & =10. \end{array}$$

Here, $N_{p}$ is the population size, $N_{t}$ is the tournament size, $p_{c}$ is the crossover probability, $p_{m}^{0}$ and $p_{m}^{+}$ are the base and intensified mutation probabilities, respectively, and τ is the stagnation threshold. The adaptive-mutation rule is enabled identically in all four variants and is therefore not treated as an ablation factor.

The four variants differ only in elite preservation and budgeted local refinement, as summarized in Table 1.

In Table 1, $N_{e}$ denotes the number of retained elite individuals, $p_{\mathrm{LS}}$ is the probability of activating local refinement for an evaluated offspring, and *L* is the maximum number of neighborhood trials per invocation. Consequently, comparisons among the four variants isolate the individual and combined effects of elite preservation and budgeted local refinement.

#### 5.1.4. FE-Fair Paired Protocol and Statistical Analysis

Algorithms with embedded local refinement may perform more objective-function evaluations within one generation than conventional evolutionary algorithms. Using the same number of generations would therefore provide an unequal computational budget. All algorithms are consequently terminated according to the same maximum number of fitness evaluations:

(86) $${\mathrm{FE}}_{\max}=30030.$$

Every call to the objective function is counted as one fitness evaluation, including evaluations during population initialization, offspring assessment, local refinement, and particle assessment. Cached fitness values of retained elite individuals are reused without consuming additional evaluations.

For each problem scale, 30 paired independent runs are conducted. Within each run, all methods use the same warehouse instance and the same instance seed. The four ablation algorithms additionally use the same initial population. Because PSO employs a different representation, it generates its own random-key swarm using the corresponding paired initialization and search seeds. Different seed sets are used across independent runs.

The reported descriptive statistics include the best value, mean value, and standard deviation of the final objective values. Relative improvement is calculated using Base-GA as the reference for the ablation study and PSO as the reference for the external comparison.

For the four-algorithm ablation study, the Friedman test with the Iman–Davenport correction is first applied to examine the overall performance difference. After rejection of the omnibus null hypothesis, paired Wilcoxon signed-rank tests with Holm correction are conducted between ED-SRMA and the three ablation baselines. Win/tie/loss counts and rank-biserial effect sizes are reported for these post hoc comparisons. All statistical tests are two-sided, with the significance level set to

(87) α=0.05.

The experiments in this study are numerical simulations based on the sensor-informed travel-time formulation. Physical sensor measurements, digital-twin validation, and deployment on an operational AS/RS platform are outside the scope of the present experiments and are discussed in Section 5.7.

### 5.2. Effect of the RS Operating Rule

To isolate the operational benefit of the RS mechanism, this subsection compares two operating modes under the same dual-I/O warehouse layout. The first mode, denoted as Dual-I/O No-RS, performs retrieval and storage actions independently. A location released by a retrieval task is added to the dynamic empty-location set, while the subsequent storage task is assigned to another available location according to the decoding rule. The second mode, denoted as Dual-I/O RS, permits an immediately following storage action to reuse the location released by the preceding retrieval operations. In this case, the two actions are completed at the same rack position, and the travel time between them is zero.

The two modes use the same ED-SRMA optimizer, warehouse instances, initial populations, paired random seeds, algorithm parameters, and fitness-evaluation budget. Therefore, the observed difference is attributable to the RS operating rule rather than to the warehouse topology or optimization method.

The relative improvement achieved by the RS mode is calculated as

(88) $$\Delta_{\mathrm{RS}}=\frac{{\bar{F}}_{\mathrm{No}\text{-}\mathrm{RS}}-{\bar{F}}_{\mathrm{RS}}}{{\bar{F}}_{\mathrm{No}\text{-}\mathrm{RS}}}\times 100\%,$$

where ${\bar{F}}_{\mathrm{No}\text{-}\mathrm{RS}}$ and ${\bar{F}}_{\mathrm{RS}}$ denote the mean total travel times obtained under the two operating modes, respectively.

**Figure 4:**
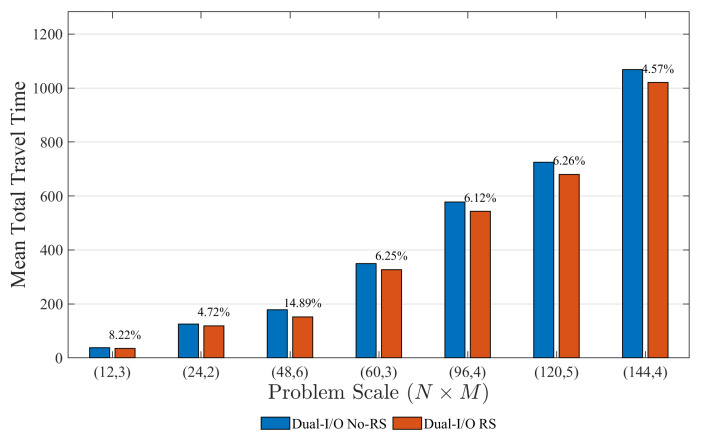
Comparison of the mean total travel times obtained under the Dual-I/O No-RS and Dual-I/O RS operating modes. The percentages above the RS bars indicate the reductions relative to the corresponding No-RS results.

As shown in Figure 4, the Dual-I/O RS mode achieves a lower mean total travel time than the Dual-I/O No-RS mode for all seven tested problem scales. The corresponding reductions are 8.22%, 4.72%, 14.89%, 6.25%, 6.12%, 6.26%, and 4.57% for the 12×3, 24×2, 48×6, 60×3, 96×4, 120×5, and 144×4 instances, respectively.

The largest improvement, 14.89%, is obtained for the 48×6 instance, where the relatively large shuttle capacity creates more opportunities to place a storage action immediately after a retrieval operation within the same trip. The 12×3 instance also exhibits a notable reduction of 8.22%. For the remaining instances, the improvement ranges from 4.57% to 6.26%, indicating that the RS mechanism provides a consistent reduction in crane travel time across different task sizes and shuttle capacities.

The benefit of the RS mode arises from the elimination of an intermediate movement between a retrieval location and a separately selected empty location. Under the No-RS mode, a retrieval operation releases its location to the empty-location set, but the subsequent storage action must still travel to an assigned empty location. Under the RS mode, the released location is reused immediately when an adjacent retrieval–storage pattern occurs. This reduces empty travel without changing the number of tasks, the dual-I/O configuration, or the fitness-evaluation budget.

The magnitude of the improvement is not monotonic with the number of requests. It depends jointly on the task sequence, shuttle capacity, spatial distribution of retrieval locations, and the frequency of feasible adjacent retrieval–storage patterns. In particular, a larger capacity can increase the number of potential RS combinations within a trip, but it also enlarges the within-trip sequencing space. Consequently, the operational gain varies across problem scales.

These results confirm that immediate location reuse consistently reduces crane travel under the tested dual-I/O configurations.

### 5.3. Ablation Study and Statistical Analysis

A controlled two-factor ablation study was conducted to quantify the individual and combined contributions of elite preservation and budgeted local refinement. Base-GA denotes the common genetic backbone with neither mechanism enabled; ED-GA enables only elite preservation; SR-MA enables only local refinement; and ED-SRMA enables both mechanisms.

Apart from these two factors, all variants use identical initialization, tournament selection, crossover, coupled and adaptive mutation, feasibility repair, cross-layer synchronization, state-dependent decoding, objective function, random settings, and fitness-evaluation budget. The mechanism configurations are summarized in Table 2.

**Figure 5:**
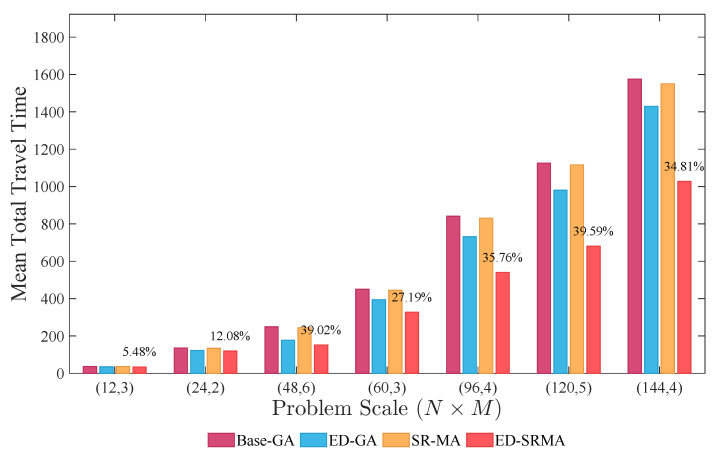
Comparison of the mean total travel times of the four algorithms across all problem scales under an identical fitness-evaluation budget. The labels above the ED-SRMA bars indicate the percentage reduction relative to Base-GA.

The performance results over 30 paired independent runs are reported in Table 3 and Figure 5. The relative improvement of algorithm *a* over Base-GA is calculated as

(89) $${\mathrm{Gap}}_{a}=\frac{{\bar{F}}_{\mathrm{Base}\text{-}\mathrm{GA}}-{\bar{F}}_{a}}{{\bar{F}}_{\mathrm{Base}\text{-}\mathrm{GA}}}\times 100\%,$$

where ${\bar{F}}_{a}$ denotes the mean final objective value of algorithm *a*.

Accordingly, any systematic performance difference among the four variants can be attributed to elite preservation, budgeted local refinement, or their interaction under the controlled experimental settings. ED-GA reduces the mean total travel time by 4.38–28.72% relative to Base-GA, showing that elite preservation provides a consistent improvement in solution quality. SR-MA achieves smaller reductions of 0.82–1.94%, indicating that locally improved solutions may be lost when no elite mechanism is used. ED-SRMA obtains the lowest mean objective value for all seven problem scales, with improvements of 5.48–39.59%. The largest reduction occurs for the 120×5 instance, where the mean total travel time decreases from 1125.87 to 680.13 and the best value decreases from 1073.00 to 638.00. Improvements of 39.02%, 35.76%, and 34.81% are also obtained for the 48×6, 96×4, and 144×4 instances, respectively.

These results indicate that elite preservation contributes more consistently than local refinement when the two mechanisms are applied independently. Their combination produces the strongest performance because locally refined solutions can be retained and propagated through subsequent population updates. The increasing performance differences on several medium- and large-scale instances further suggest that the combined mechanism is particularly effective when shuttle capacity enlarges the within-trip sequencing space.

To determine whether the observed differences were attributable to random variation, a Friedman test was applied to the final objective values over the 210 paired scale–run blocks. The null hypothesis of equivalent performance was rejected:

(90) $$\chi_{F}^{2}\left(3\right)=552.1433,\;p=2.3842\times 10^{-119}.$$

The corresponding Iman–Davenport statistic was

(91) $$F_{\mathrm{ID}}\left(3,627\right)=1482.1849,\;p<0.001.$$

Following the significant omnibus result, paired Wilcoxon signed-rank tests with Holm correction were conducted between ED-SRMA and each ablation baseline. The results are reported once in Table 4.

Almost all Holm-adjusted *p*-values are below 0.001, and the rank-biserial effect sizes range from 0.9769 to 1.0000, indicating statistically significant and large effects in favor of ED-SRMA. The median improvements over Base-GA, ED-GA, and SR-MA are 34.04%, 18.19%, and 32.73%, respectively. For the smallest 12×3 instance, however, the difference between ED-SRMA and ED-GA is not significant after Holm correction ($p_{\mathrm{Holm}}=0.0911$), because the restricted search space allows both methods to obtain similar solutions. The advantage of the complete framework becomes clearer as the scheduling and within-trip sequencing complexity increase.

### 5.4. External Comparison with Particle Swarm Optimization

Particle swarm optimization (PSO) was selected as an external benchmark to contrast a continuous random-key metaheuristic against the problem-specific discrete representation of ED-SRMA. PSO represents candidate schedules using continuous random keys, which are deterministically decoded into retrieval-task assignments and within-trip operation sequences. Both algorithms use the same warehouse instances, population size, objective function, and fitness-evaluation budget defined in Equation (86) over 30 paired independent runs. All evaluations generated by the local-refinement procedure of ED-SRMA are included in this budget.

The relative improvement of ED-SRMA over PSO is defined as

(92) $$\Delta_{\mathrm{PSO}}=\frac{{\bar{F}}_{\mathrm{PSO}}-{\bar{F}}_{\mathrm{ED}\text{-}\mathrm{SRMA}}}{{\bar{F}}_{\mathrm{PSO}}}\times 100\%,$$

where ${\bar{F}}_{\mathrm{PSO}}$ and ${\bar{F}}_{\mathrm{ED}\text{-}\mathrm{SRMA}}$ denote the corresponding mean final total travel times.

**Figure 6:**
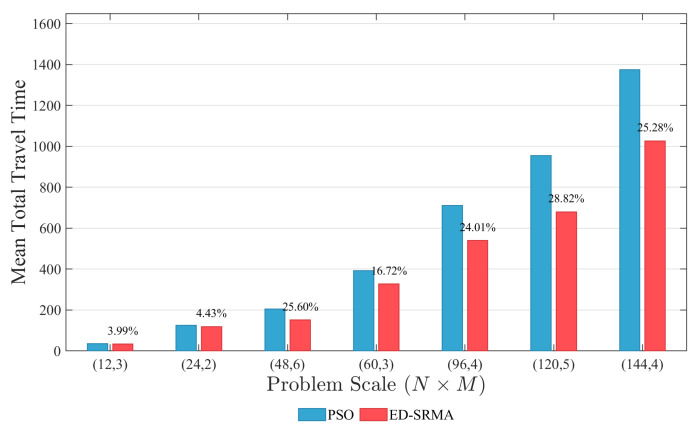
Comparison of the mean total travel times obtained by PSO and ED-SRMA across seven problem scales under the same fitness-evaluation budget. The percentages above the ED-SRMA bars denote the reductions relative to PSO.

As shown in Figure 6, ED-SRMA obtains lower mean total travel times than PSO for all seven tested instances. The reductions are 3.99%, 4.43%, 25.60%, 16.72%, 24.01%, 28.82%, and 25.28% for the 12×3, 24×2, 48×6, 60×3, 96×4, 120×5, and 144×4 instances, respectively. The differences are small for the two smallest instances but become more pronounced for the medium- and large-scale problems. The largest improvement, 28.82%, is obtained for the 120×5 instance.

The stronger performance on the more complex instances can be attributed to the problem-specific representation and constraint-handling procedures of ED-SRMA. Its dual-layer structure separates task assignment from operation sequencing, while repair and synchronization ensure that only feasible and internally consistent schedules are evaluated. Elite preservation and local refinement further retain and improve promising solutions. By contrast, PSO searches in a continuous random-key space and depends on decoding to construct discrete schedules.

### 5.5. FE-Based Convergence Analysis

**Figure 7:**
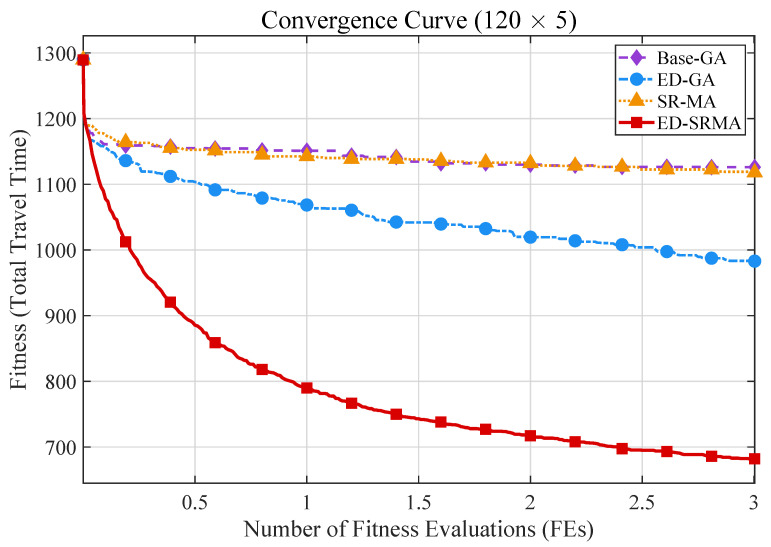
FE-based convergence comparison of the four ablation algorithms for the 120×5 instance. Each curve represents the median best-so-far objective value over 30 paired independent runs under a common budget of 30,030 fitness evaluations.

To examine the convergence behavior of the four ablation algorithms under a fair computational budget, the 120×5 instance was selected as a representative case. This instance contains a relatively large number of tasks and a complex within-trip sequencing structure. Figure 7 shows the median best-so-far objective values obtained by Base-GA, ED-GA, SR-MA, and ED-SRMA over 30 paired independent runs.

Within each paired run, the four algorithms use the same problem instance and initial population. The horizontal axis represents the cumulative number of fitness evaluations rather than generations. All objective-function calls, including those performed during population initialization, offspring evaluation, and local refinement, are counted toward the common budget of 30,030 fitness evaluations.

As shown in Figure 7, the four algorithms begin from similar objective-value ranges because they share the same initial population. ED-SRMA produces a more rapid reduction during the early stage, decreasing below approximately 900 within the first 5000 fitness evaluations. Its best-so-far objective value continues to decrease over the remaining budget and reaches approximately 680 at termination.

ED-GA also improves progressively but stabilizes at a higher objective value of approximately 980. Base-GA and SR-MA obtain most of their improvements during the early stage and subsequently remain near 1125 and 1120, respectively. These convergence patterns are consistent with the final solution-quality results reported in Section 5.3.

The comparison further illustrates the complementary contributions of the mechanisms incorporated into ED-SRMA. Elite preservation prevents promising solutions from being discarded during population replacement, whereas the budgeted local-refinement procedure provides additional opportunities to improve selected feasible offspring. ED-GA benefits from elite preservation but does not include neighborhood refinement. In contrast, SR-MA applies local refinement without explicitly retaining elite individuals, so improvements obtained by individual offspring may not be preserved during subsequent population updates.

Under the common fitness-evaluation budget, ED-SRMA therefore achieves both an earlier reduction in the objective value and a lower final total travel time for the tested instance. The result indicates that the combined framework uses the available objective evaluations more effectively than the three ablation variants, although the conclusion is restricted to the examined instance and experimental settings.

### 5.6. Sensitivity Analysis of Shuttle Capacity

**Figure 8:**
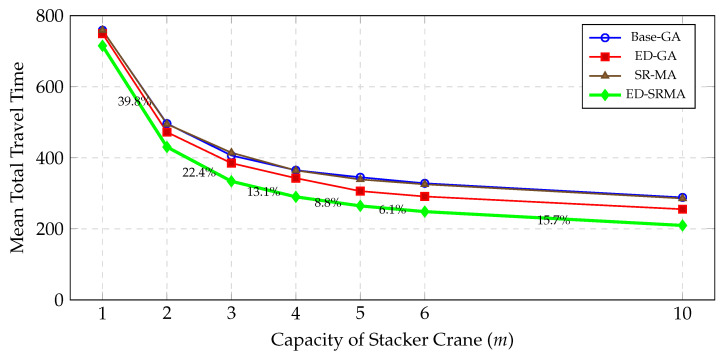
Capacity sensitivity analysis: the decrease rate between adjacent shuttle capacities is annotated for ED-SRMA, illustrating diminishing marginal returns as the shuttle capacity *m* increases (n=60).

The shuttle capacity *m*, which denotes the number of unit loads a crane can carry simultaneously, is a critical hardware parameter that significantly affects the operational efficiency of the AS/RS. In this section, we examine the impact of *m* on the total travel time by evaluating seven capacity settings ranging from 1 to 10 while keeping the total request size constant at n=60. The experimental results are presented in Figure 8.

As shown in Figure 8, there is a clear negative correlation between the shuttle capacity *m* and the total travel time. Increasing *m* allows the crane to consolidate more storage and retrieval tasks into a single trip, thereby reducing the frequency of empty return trips to the I/O ports and enhancing the utilization of RS operations. However, a phenomenon of diminishing marginal returns is observed: the most significant reductions in travel time occur when *m* increases from 1 to 4. When *m* exceeds 6, the performance curve begins to plateau, and the marginal benefit of further increasing capacity becomes negligible. This suggests that, for the tested warehouse scale, a capacity of m∈[4,6] represents an optimal trade-off between hardware cost and operational throughput.

A noteworthy observation is that the performance gap between the proposed ED-SRMA and the Base-GA widens significantly as *m* increases. When the shuttle capacity is large, the number of possible task sequences and interleaved storage/retrieval patterns within each trip grows exponentially. Specifically, for a trip with capacity *m*, the theoretical number of operation sequences is related to (2m)!, leading to a combinatorial explosion in the solution space. Under these conditions, the Base-GA often fails to identify high-quality sequences due to the lack of specialized search pressure, resulting in stagnant performance at high *m* values. In contrast, the ED-SRMA leverages its memetic local search and synchronized repair operators to effectively prune the search space and refine complex operation sequences. This capability allows ED-SRMA to maintain a steep efficiency improvement curve even under high loads, demonstrating its robust optimization capability for high-dimensional combinatorial problems.

### 5.7. Discussion and Limitations of Numerical Validation

#### 5.7.1. Discussion of the Experimental Findings

Taken together, the experiments provide complementary evidence regarding the operating rule and the proposed solution method. The comparison under the same dual-I/O configuration confirms that immediate reuse of a released retrieval location can reduce unnecessary crane travel. The ablation study further shows that elite preservation and budgeted local refinement play complementary roles: local refinement improves selected feasible solutions, while elite preservation enables these improvements to be retained during subsequent population updates. The comparison with PSO indicates that the observed advantage is not limited to closely related genetic variants, and the FE-based convergence analysis for the representative 120×5 instance shows that ED-SRMA uses the common evaluation budget more effectively.

The performance advantage of ED-SRMA generally becomes more evident when larger task sets or shuttle capacities enlarge the feasible sequencing space. At the same time, increasing shuttle capacity reduces the number of crane trips but provides diminishing marginal reductions in travel time. These findings indicate that warehouse performance depends jointly on equipment capacity, operating rules, and scheduling quality.

#### 5.7.2. Limitations and Scope of Validation

The conclusions above should be interpreted within the scope of the numerical experiments. First, the experiments use synthetically generated warehouse instances and a sensor-informed travel-time model. No measurements from an operational AS/RS, digital-twin platform, or physical prototype are used to validate the predicted travel times. The displacement and I/O-position sensors described in the model therefore represent the intended data interface for future deployment rather than a source of experimental measurements in the present study.

Second, the model assumes a single crane, known task locations, deterministic task availability, constant axis velocities, homogeneous handling times, and full-load trips. Acceleration, deceleration, mechanical delays, energy consumption, unexpected equipment states, dynamic order arrivals, and interference among multiple cranes are not considered. These assumptions make it possible to isolate the scheduling mechanisms but limit direct generalization to more complex industrial environments.

Third, the algorithmic results are based on the selected problem scales, parameter settings, and fitness-evaluation budget. Although paired runs and nonparametric tests reduce the influence of random variation, they do not establish universal superiority over all possible instances or optimization methods. Runtime values are also dependent on the computing platform and implementation details. In addition, PSO is used as a representative external metaheuristic rather than as an exhaustive comparison with all available optimization approaches.

## 6. Conclusions

This study shows that jointly considering storage-location allocation, storage/retrieval sequencing, dynamic location reuse, and inter-trip I/O continuity can substantially reduce crane travel in a dual-I/O shared-storage AS/RS. The proposed ED-SRMA was used to solve this integrated scheduling problem under a common fitness-evaluation budget.

The numerical results lead to three main findings. First, immediate reuse of retrieval locations through the RS operating rule reduced the mean total travel time by 4.57–14.89% relative to the corresponding dual-I/O No-RS mode, confirming that dynamic location reuse provides a measurable operational benefit. Second, ED-SRMA achieved the lowest mean objective value at all seven tested scales, with reductions of 5.48–39.59% relative to Base-GA and 3.99–28.82% relative to PSO. Third, the ablation results indicate that elite preservation and budgeted local refinement are complementary: local refinement improves selected feasible solutions, whereas elite preservation helps retain these improvements during subsequent population updates. The Friedman and paired Wilcoxon tests with Holm correction confirmed significant differences in the ablation comparisons, with adjusted *p*-values below 0.001 and rank-biserial effect sizes of 0.9769–1.0000. For the representative 120×5 instance, ED-SRMA also exhibited an earlier reduction in the objective value and continued improvement under the common fitness-evaluation budget.

These findings suggest that the performance of dual-I/O shared-storage systems depends not only on equipment capacity, but also on whether dynamic storage-location changes and I/O-state transitions are explicitly incorporated into scheduling decisions. The increasing advantage observed for several medium- and large-scale instances further indicates that problem-specific feasibility restoration and solution retention become more important as the within-trip sequencing space expands. The sensor-informed travel-time formulation also provides a direct interface through which analytical schedules may subsequently be evaluated using measured crane-motion data.

The conclusions remain limited to numerical experiments conducted under simplified and deterministic operating assumptions. Their generalizability to industrial systems with realistic motion dynamics, uncertain order arrivals, multiple interacting cranes, and heterogeneous handling conditions has not yet been established. Future research will therefore focus on validation using measured data or a digital-twin platform, extensions to dynamic and multi-crane environments, and comparisons with exact and additional metaheuristic methods on broader industrial benchmark sets. In addition, a trajectory-level analysis will be conducted by recording the accepted and rejected evolutionary and local-refinement moves and mapping them to transitions among attraction basins. Local-optima-network and search-trajectory-network analyses [41,42] could then be used to distinguish successful, unsuccessful, deceptive, and rejected search behaviors, thereby providing a more precise explanation of why particular operators succeed or fail at different stages of the search. 

## Figures and Tables

**Table 1:** Controlled mechanism settings of the four ablation variants.

Algorithm	Elite Preservation	$N_{e}$	Local Refinement	$p_{\mathrm{LS}}$	*L*
Base-GA	No	0	No	0	0
ED-GA	Yes	2	No	0	0
SR-MA	No	0	Yes	0.20	3
ED-SRMA	Yes	2	Yes	0.20	3

**Table 2:** Mechanism configuration of the ablation algorithms.

Algorithm	Elite-Driven Update	Local Refinement
Base-GA	No	No
ED-GA	Yes	No
SR-MA	No	Yes
ED-SRMA	Yes	Yes

**Table 3:** Performance comparison of the four algorithms under an identical budget of 30,030 fitness evaluations. Best, Mean, and Std Dev are calculated over 30 independent runs, and Gap denotes the percentage reduction in the mean total travel time relative to Base-GA.

n×m	Algorithm	Best	Mean	Std Dev	Gap (%)
12×3	Base-GA	31.00	36.50	2.0129	–
	ED-GA	29.00	34.90	2.0569	4.38%
	SR-MA	30.00	35.97	1.9911	1.46%
	ED-SRMA	29.00	34.50	2.0968	5.48%
24×2	Base-GA	124.00	135.80	5.9038	–
	ED-GA	109.00	122.37	7.2848	9.89%
	SR-MA	122.00	134.17	6.2977	1.20%
	ED-SRMA	106.00	119.40	7.1032	12.08%
48×6	Base-GA	238.00	249.33	6.5933	–
	ED-GA	165.00	177.73	8.5980	28.72%
	SR-MA	229.00	244.50	9.0620	1.94%
	ED-SRMA	142.00	152.03	5.5985	39.02%
60×3	Base-GA	421.00	449.83	14.0984	–
	ED-GA	361.00	394.67	18.6276	12.26%
	SR-MA	422.00	444.80	11.8857	1.12%
	ED-SRMA	307.00	327.53	12.9475	27.19%
96×4	Base-GA	809.00	841.37	17.6449	–
	ED-GA	678.00	731.03	28.0977	13.11%
	SR-MA	802.00	831.43	16.6892	1.18%
	ED-SRMA	511.00	540.47	16.7738	35.76%
120×5	Base-GA	1073.00	1125.87	22.0372	–
	ED-GA	905.00	980.47	36.7768	12.91%
	SR-MA	1063.00	1116.63	24.9613	0.82%
	ED-SRMA	638.00	680.13	19.4648	39.59%
144×4	Base-GA	1515.00	1575.67	31.8957	–
	ED-GA	1324.00	1430.00	47.4116	9.24%
	SR-MA	1484.00	1550.47	34.7699	1.60%
	ED-SRMA	975.00	1027.20	23.1180	34.81%

**Table 4:** Statistical analysis of the ablation study using paired Wilcoxon signed-rank tests with Holm correction.

Comparison	W/T/L	Holm-Adjusted *p*	Rank-Biserial Effect Size	Median Improvement (%)
ED-SRMA vs. Base-GA	207/3/0	<0.001	1.0000	34.04
ED-SRMA vs. ED-GA	186/13/11	<0.001	0.9769	18.19
ED-SRMA vs. SR-MA	206/3/1	<0.001	0.9995	32.73

## Data Availability

The data presented in this study are available on request from the corresponding author.
